# The role of isoniazid dosage and NAT2 gene polymorphism in the treatment of tuberculous meningitis

**DOI:** 10.3389/fimmu.2024.1535447

**Published:** 2025-01-20

**Authors:** Yi Jian, Yuhang Bao, Fashuang Yang, Mei Zhu

**Affiliations:** ^1^ Department of Hematology, The Affiliated Hospital of Zunyi Medical University, Zunyi, Guizhou, China; ^2^ Department of Respiratory and Critical Care Medicine, The People’s Hospital of Zhijin County, Bijie, Guizhou, China; ^3^ Department of Gastroenterology, The People’s Hospital of Jinsha County, Bijie, Guizhou, China; ^4^ Department of Respiratory and Critical Care Medicine, Zhejiang Provincial People’s Hospital Bijie Hospital, Bijie, Guizhou, China

**Keywords:** tuberculous meningitis, NAT2, isoniazid, treatment, prognosis

## Abstract

**Background:**

Tuberculous meningitis (TBM) is a non-purulent inflammatory condition affecting the meninges and spinal membranes, caused by Mycobacterium tuberculosis (MTB) infection. This study seeks to explore the impact of varying INH dosages and NAT2 gene polymorphisms on TBM treatment, contributing new insights to improve clinical management and patient prognosis.

**Methods:**

Patients with TBM hospitalized between July 2020 and December 2022 were categorized into two groups based on INH dosage: the standard-dose group (300 mg/day) and the high-dose group (600 mg/day). General and baseline data were collected, and NAT2 genotypes were identified using real-time fluorescent PCR with melting curve analysis. The clinical characteristics and outcomes of patients with TBM under varying INH dosages were analyzed.

**Results:**

This study enrolled 119 patients with TBM, including 32 (26.9%) in the standard-dose group and 87 (73.1%) in the high-dose group. The NAT2 genotypes were distributed as follows: 34 (28.6%) fast acetylators (FA), 73 (61.3%) intermediate acetylators (IA), and 12 (10.1%) slow acetylators (SA). By month 12, 25 patients (21.0%) experienced disability or death, with 22 cases (18.5%) occurring by the end of the 3rd month. Disability and mortality rates differed significantly between the standard-dose and high-dose groups for IA-type TBM patients (P = 0.014). Univariate analysis showed significant differences between groups in baseline focal neurological impairment and disability or mortality by the 3rd and 12th months. Multivariate logistic regression identified INH dosage, cranial nerve palsy, age, and headache as key prognostic factors for TBM.

**Conclusion:**

High-dose INH treatment was associated with a reduced incidence of disability or death compared to the standard-dose regimen, indicating better efficacy and prognosis. In patients with IA-type TBM, the high-dose group showed a significantly lower rate of disability or mortality, suggesting that higher INH dosages may reduce the risk of adverse outcomes.

## 1 Introduction

Tuberculous meningitis (TBM) is a non-purulent inflammatory condition that affects the meninges and spinal membranes, caused by infection with Mycobacterium tuberculosis (MTB). It represents the most common form of central nervous system tuberculosis (1–3) and is associated with the highest rates of disability and mortality among all forms of tuberculosis (4, 5). Isoniazid (INH), also known as isonicotinic acid hydrazide, is a key antibiotic in the treatment of tuberculosis, renowned for its potent early bactericidal activity and its ability to penetrate the meninges, making it essential in the management of TBM.

The metabolic rate of INH in the human body is influenced by the activity of N-acetyltransferase 2 (NAT2) (6, 7). NAT2 is a pivotal phase II detoxification enzyme that catalyzes the transfer of acetyl groups from acetyl-CoA to aromatic and heterocyclic amines, playing a crucial role in the activation and/or inactivation of these compounds, as well as in the metabolism of various drugs. NAT2 is expressed in the epithelium of several organs, including the esophagus, stomach, small intestine, large intestine, and bladder, with the liver and intestinal epithelial cells being the primary sites of expression. In 1995, Bell et al. identified polymorphisms in the NAT2 gene, which were shown to significantly affect the enzyme’s expression, stability, and catalytic activity. Based on these polymorphisms, individuals can be classified as fast acetylators (FA), intermediate acetylators (IA), or slow acetylators (SA) (8). Plasma drug concentrations differ significantly among individuals with various acetylation phenotypes when the same INH dose is administered. In China, the recommended INH dosage typically ranges from 300 to 600 mg/day.

The optimal clinical dosage of INH remains controversial. The standard adult dose of INH is generally capped at 300 mg/day. However, prior studies suggest that increasing the dose to 600–900 mg/day could enhance treatment efficacy and reduce the risk of developing drug-resistant tuberculosis. Therapeutic outcomes in patients with TBM may be closely linked to both INH dosage and NAT2 gene polymorphism (9). Tailoring INH dosage based on NAT2 polymorphism may optimize treatment regimens, potentially minimizing treatment failures and adverse effects (10). This study seeks to explore the impact of varying INH dosages and NAT2 gene polymorphisms on TBM treatment, contributing new insights to improve clinical management and patient prognosis.

## 2 Materials and methods

### 2.1 Patient inclusion and exclusion

The Ethics Committee of the Affiliated Hospital of Zunyi Medical University approved this study (Ethics Approval Number: KLLY-2021-022). This study retrospectively included 119 patients diagnosed with TBM who were hospitalized and treated at our institution between July 2020 and December 2022. Clinical data were obtained through a comprehensive review of electronic medical records. The diagnosis of TBM was made according to the criteria established by Suzaan Marais et al. (5). Patients were classified into three categories based on the diagnostic criteria: confirmed, highly probable, or possible cases. Confirmed cases fulfilled the clinical inclusion criteria and also met one or more of the following: (a) identification of antibiotic-resistant bacteria in cerebrospinal fluid (CSF), (b) positive MTB culture from CSF, or (c) detection of MTB nucleic acid in CSF. Highly probable cases met the clinical inclusion criteria and had a diagnostic score of ≥12 (or ≥10 in the absence of imaging), with at least 2 points derived from either CSF analysis or cranial imaging, and other differential diagnoses were excluded. Possible cases met the clinical inclusion criteria and had a diagnostic score between 6 and 11 points (or 6-9 in the absence of imaging); these cases did not undergo lumbar puncture or cranial imaging and could not be definitively confirmed or excluded as TBM.

Exclusion criteria encompassed a history of prior anti-tuberculosis treatment, intolerance to first-line anti-tuberculosis drugs, initiation of treatment more than one week before hospital admission, resistance to isoniazid (INH) or rifampin detected before or during treatment, elevated alanine aminotransferase (ALT) or creatinine levels beyond the upper limit of normal at treatment initiation, long-term use of immunosuppressive agents, severe underlying comorbidities, pregnancy or lactation, and other factors necessitating exclusion. These stringent criteria were applied to ensure the selection of a homogenous study population for assessing the influence of INH dosage and NAT2 gene polymorphism on the treatment outcomes of TBM. The patients were divided into two groups based on the prescribed INH dosage: the standard-dose group (300 mg/day) and the high-dose group (600 mg/day), comprising 32 patients and 78 patients, respectively.

### 2.2 Observation indicators and clinical data collection

The primary endpoint of this study was disability or mortality, while secondary endpoints included changes in clinical manifestations, laboratory parameters, and imaging findings. Comprehensive demographic data were collected for all participants, including gender, age, height, weight, smoking and alcohol use history, comorbidities, and disease duration. Clinical manifestations were systematically assessed, encompassing symptoms such as fever, headache, nausea, vomiting, cough, sputum production, weight loss, night sweats, focal neurological deficits, cranial nerve palsy, and altered mental status. Laboratory investigations included measurements of various parameters, including total white blood cell count, hemoglobin, blood glucose levels, lymphocyte count, and cerebrospinal fluid (CSF) characteristics, such as intracranial pressure, white blood cell count, lymphocyte count, protein concentration, chloride concentration, adenosine deaminase activity, and CSF-to-blood glucose ratio.

Microbiological diagnostics for Mycobacterium tuberculosis were performed through acid-fast staining of CSF, sputum, and bronchoalveolar lavage fluid, complemented by molecular assays such as Xpert MTB/RIF, LAMP, metagenomic next-generation sequencing (mNGS), and mycobacterial culture. Imaging studies included cranial and chest imaging, as well as examination of other relevant anatomical regions. The severity of TBM was assessed using the Glasgow Coma Scale (GCS) and the Medical Research Council (MRC) staging system (5).

### 2.3 Sample collection and processing

Peripheral blood DNA from patients with TBM was extracted using the Lad-Aid824s blood genomic DNA extraction reagent (Xiamen Zhishan Biotechnology Co., Ltd.). Initially, a dithiothreitol (DTT) solution (1 mol/L) was prepared by dissolving 0.45 g of DTT in deionized water to a final volume of 3000 μL. The solution was mixed thoroughly, aliquoted into small portions, and stored at temperatures below -18°C to maintain stability. Subsequently, the Lab-Aid 824s/808 nucleic acid extraction instrument was powered on, and the initial interface appeared on the display screen. Reagent strips were placed on the instrument tray, and the sealing membrane was removed. Then, 200–500 μL of blood cell samples and 60 μL of the prepared DTT solution were added to the first well of each reagent strip. The tray was inserted into the instrument base and pushed into the instrument chamber. The “NA-DNA” program was selected, and the “Start” button was pressed to initiate the automated extraction process. Upon completion of the program, the tray was removed, and the eluted DNA was transferred to a centrifuge tube using a pipette. Finally, the concentration and purity of the extracted DNA were evaluated using a UV spectrophotometer. The acceptable range for the OD 260/OD 280 ratio was between 1.6 and 2.0, and the OD 260/OD 230 ratio was required to be ≥ 2.0. NAT2 genotyping was subsequently performed using real-time PCR combined with melting curve analysis.

### 2.4 Treatment and follow-up

All enrolled patients received treatment in accordance with the “2019 Chinese Central Nervous System Tuberculosis Diagnosis and Treatment Guidelines,” as issued by the Tuberculosis and Mycobacterial Diseases (TBM) Professional Committee of the Chinese Medical Association in July 2020. The anti-tuberculosis chemotherapy regimen consisted of a 3-month intensive phase with isoniazid, rifampin, pyrazinamide, and ethambutol (3HRZE), followed by a 9-month continuation phase with isoniazid and rifampin (9HR). The administration and duration of corticosteroid therapy adhered to the “2015 Chinese Tuberculosis Diagnosis and Treatment Guidelines” issued by the Chinese Medical Association. Follow-up was conducted through a comprehensive review of hospitalization records, outpatient consultations, and phone interviews. Data collected during follow-up included patients’ clinical symptoms post-discharge, subsequent treatments, prognosis, and survival status. The follow-up period ranged from 1 to 12 months, with a cutoff date of December 30, 2022. All patients successfully completed the follow-up process.

### 2.5 Statistical methods

Statistical analyses were performed using SPSS 18.0 software. Categorical data were presented as [n(%)]. Continuous data were first tested for normality. Normally distributed data were expressed as mean ± SD, while non-normally distributed data were reported as median (*P*25, *P*75). Differences in categorical data between groups were analyzed using the chi-square test or Fisher’s exact test. For normally distributed continuous data, differences between two groups were compared using the independent sample t-test. For non-normally distributed continuous data, the *M*ann-Whitney *U* test was used. A significance level of α = 0.05 was set, with *P* < 0.05 considered statistically significant.

## 3 Results

### 3.1 General demographic information

**Figure 1:**
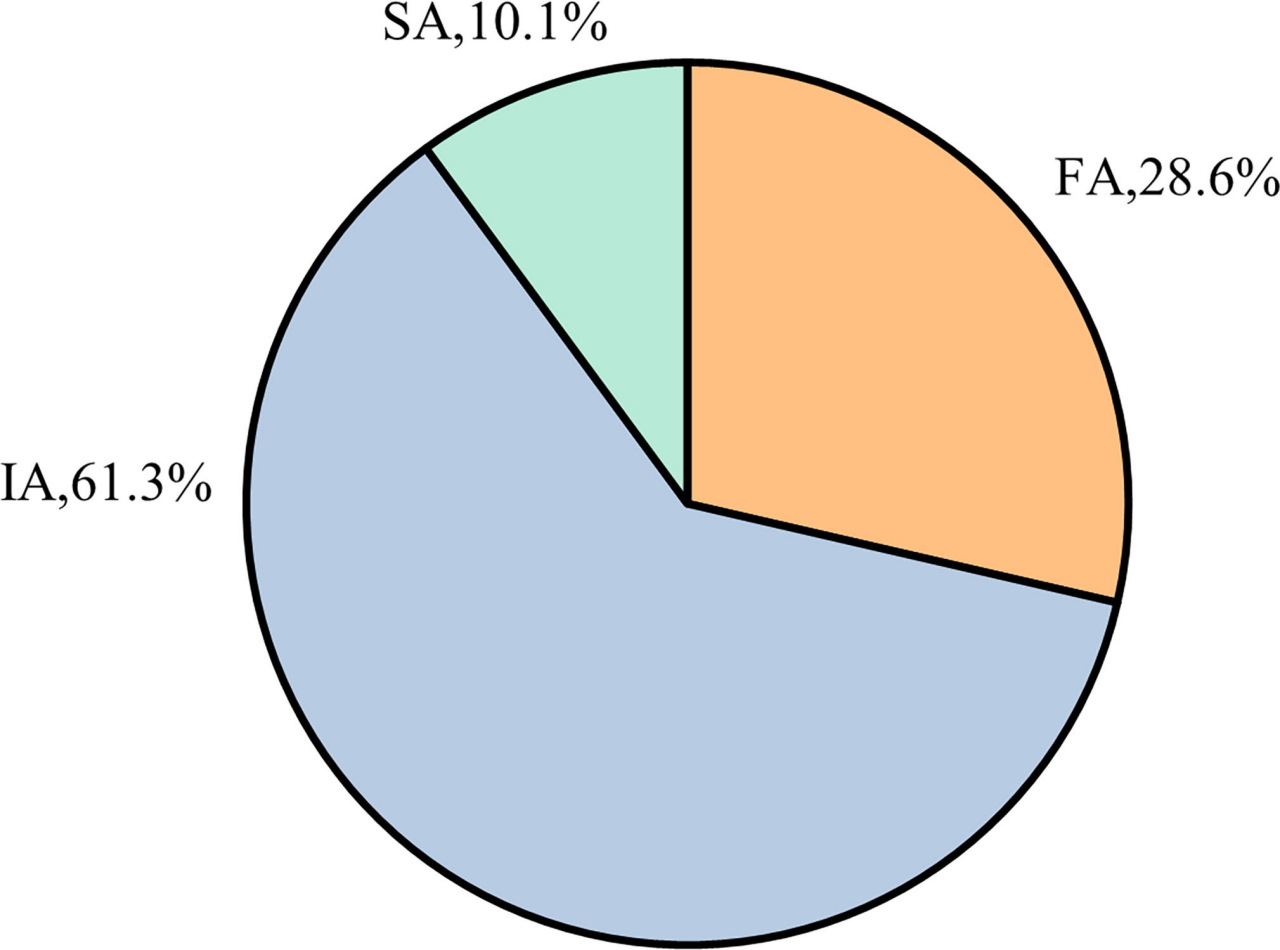
Distribution of NAT2 genotypes among included patients with TBM.

Based on the inclusion and exclusion criteria, a total of 119 patients with TBM who met the eligibility requirements were selected. Among these patients, the distribution of NAT2 gene polymorphisms was as follows: the IA type represented the largest proportion (approximately 61.3%), followed by the FA type (28.6%) and the SA type (10.1%), as shown in **Figure 1**.

The cohort included 119 patients with TBM, with a higher proportion of males than females. The mean age was 42.13 ± 19.21 years, and the average body weight was 52.57 ± 7.96 kg. The mean disease duration was approximately 29.14 ± 44.45 days. Among the patients, 42% had comorbidities. Regarding the clinical classification, 34.5% were classified as confirmed cases, 35.3% as highly probable cases, and 30.3% as probable cases. The distribution of NAT2 genotypes was predominantly IA type, followed by FA and SA types, with IA type being the most common. Of the total patients, 32 (26.9%) were in the standard-dose group, while 87 (73.1%) were in the high-dose group. Detailed information is presented in **Table 1**.

**Table 1:** Comparison of basic information between standard-dose and high-dose groups.

Indicator	Standard-Dose Group (N=32)	High-Dose Group (N=87)	Statistic	*P*
Gender	Male 17 (53.1)	Male 45 (51.7)	χ²=0.018	0.892
Age (years)	47.50 (30.00,65.00)	37.00 (23.00,57.00)	Z=-1.601	0.109
Weight (kg)	50.00 (45.75,55.75)	51.00(48.00,59.75)	Z=-0.249	0.804
Disease Duration (day)	11.00 (7.75,30.00)	12.00 (7.00,30.00)	Z=-0.472	0.637
Smoking	No 21 (65.6)	No 60 (69.0)	χ²=0.120	0.729
Drinking	No 24 (75.0)	No 64 (73.6)	χ²=0.025	0.874
Comorbidities	No 17 (53.1)	No 52 (59.8)	χ²=0.424	0.515

### 3.2 Differences in baseline characteristics

The baseline demographic data of patients with tuberculous meningitis (TBM), including gender, age, weight, disease duration, smoking history, alcohol consumption, and comorbidities, revealed no statistically significant differences between the standard-dose and high-dose treatment groups (P > 0.05), as summarized in **Table 1**. The distribution of TBM manifestations at baseline showed that fever was present in 81 cases (68.1%), headache in 90 cases (75.6%), and nausea in 52 cases (43.7%). Other common symptoms included vomiting (49 cases, 41.2%), cough (53 cases, 44.5%), weight loss (21 cases, 17.6%), and night sweats (30 cases, 25.2%). Headache was the most prevalent symptom. However, no significant differences were found in the occurrence of fever, headache, nausea, vomiting, cough, weight loss, or night sweats between the two treatment groups (P > 0.05). These findings are further detailed in **Table 2**.

**Table 2:** Comparison of baseline clinical symptoms between the standard-dose and high-dose groups.

Indicator	Category	Standard-Dose Group (N=32)	High-Dose Group (N=87)	Statistic (χ²)	*P*
Fever	No	10 (31.2)	28 (32.2)	0.009	0.923
	Yes	22 (68.8)	59 (67.8)		
Headache	No	9 (28.1)	20 (23.0)	0.335	0.563
	Yes	23 (71.9)	67 (77.0)		
Nausea	No	20 (62.5)	47 (54.0)	0.683	0.408
	Yes	12 (37.5)	40 (46.0)		
Vomiting	No	22 (68.8)	48 (55.2)	1.781	0.182
	Yes	10 (31.2)	39 (44.8)		
Cough	No	17 (53.1)	49 (56.3)	0.097	0.756
	Yes	15 (46.9)	38 (43.7)		
Weight Loss	No	25 (78.1)	73 (83.9)	0.538	0.463
	Yes	7 (21.9)	14 (16.1)		
Night Sweats	No	21 (65.6)	68 (78.2)	1.950	0.163
	Yes	11 (34.4)	19 (21.8)		

### 3.3 Neurological manifestations and disease severity

Neurological manifestations were observed in a subset of patients, with 13 cases (10.9%) presenting with damage to the Stovea channel, 21 cases (17.6%) with cranial nerve palsy, and 33 cases (27.7%) with disability. Additionally, 32 patients exhibited altered consciousness, including 11 cases (9.2%) in a coma, 9 cases (7.6%) with hazy consciousness, 7 cases (5.9%) of lethargy, 3 cases (2.5%) of drowsiness, and 2 cases (1.7%) of delirium. Meningeal irritation was observed in 41 cases (34.5%). The severity of meningitis was graded as stage I in 83 cases (69.7%), and 83 cases (69.7%) had a Glasgow Coma Scale (GCS) score ≥ 15. Notably, damage to the Vesta meridian was significantly higher in the standard-dose group (21.9%) compared to the high-dose group (6.9%) (P = 0.046). However, cranial nerve palsy, altered states of consciousness, and meningeal irritation did not show significant differences between the groups (P > 0.05). Similarly, no significant differences were found in the severity of meningitis or GCS scores between the standard-dose and high-dose groups (P > 0.05). These findings are presented in **Table 3**.

**Table 3:** Comparison of baseline signs between the standard-dose and high-dose groups .

Indicator	Category	Standard-Dose Group (N=32)	High-Dose Group (N=87)	Statistic	*P*
Focal Neurological Deficits	No	25 (78.1)	81 (93.1)	**χ²**=3.964	0.046
	Yes	7 (21.9)	6 (6.9)		
Cranial Nerve Palsy	No	27 (84.4)	71 (81.6)	χ²=0.123	0.726
	Yes	5 (15.6)	16 (18.4)		
Altered Consciousness	No	25 (78.1)	62 (71.3)	χ²=2.768	0.759
	Drowsines	1 (3.1)	2 (2.3)		
	Confusion	1 (3.1)	8 (9.2)		
	Stupor	2 (6.3)	5 (5.7)		
	Coma	2 (6.3)	9 (10.3)		
	Delirium	1 (3.1)	1 (1.1)		
Meningeal Irritation Signs	No	20 (62.5)	58 (66.7)	χ²=0.180	0.672
	Yes	12 (37.5)	29 (33.3)		
Severity Staging of Meningitis	Stage I	24 (75.0)	59 (67.8)	Z=-1.097	0.272
	Stage II	6 (18.8)	9 (10.3)		
	Stage III	2 (6.3)	19 (21.8)		
GCS	15	24 (75.0)	59 (67.8)	Z=-0.969	0.333
	12-14	3 (9.4)	6 (6.9)		
	9-11	3 (9.4)	8 (9.2)		
	3-8	2 (6.3)	14 (16.1)		

### 3.4 CSF and imaging findings between groups

The analysis of cerebrospinal fluid (CSF) indicators, including intracranial pressure, white blood cell count, lymphocyte percentage, protein concentration, adenosine deaminase (ADA), CSF-to-plasma glucose ratio, glucose concentration, and chloride concentration, revealed no significant differences between the standard-dose and high-dose groups (P > 0.05). Additionally, no statistically significant differences were observed in the baseline CSF or pulmonary pathogen positivity rates between the two groups (P > 0.05) (**Table 4**). Imaging results at baseline showed hydrocephalus in 22 cases (19.8%), enhanced anterior basal cistern density in 4 cases (3.6%), basal cistern meningeal enhancement in 26 cases (23.4%), tuberculomas in 27 cases (24.3%), and infarction in 28 cases (25.2%). Chest CT scans revealed secondary pulmonary tuberculosis in 62 cases (52.1%) and hematogenous disseminated pulmonary tuberculosis in 32 cases (26.9%). Imaging findings in other body parts, aside from the brain and lungs, were noted in 18 cases (15.1%). No significant differences were found in head imaging, chest CT, or imaging of other body parts between the standard-dose and high-dose groups (P > 0.05). These results are outlined in **Table 5**.

**Table 4:** Comparison of baseline CSF analysis between the standard-dose and high-dose groups.

Indicator	Category	Standard-Dose Group (N=32)	High-Dose Group (N=87)	Statistic	*P*
Intracranial Pressure		193.21 ± 63.19	216.52 ± 91.79	t=-1.468	0.147
White Blood Cell Count		100.50 (4.25,272.50)	97.50 (20.00,326.25)	Z=-0.684	0.494
Lymphocyte Percentage		52.00 (28.75,77.00)	60.00(29.00,90.00)	Z=-0.917	0.359
Protein Concentration		1323.50(547.00,3061.50)	1358.00 (912.25,2569.75)	Z=-0.336	0.737
ADA		4.86 (0.99,8.54)	5.18 (2.64,7.92)	Z=-0.926	0.354
CSF-to-Plasma Glucose Ratio	<50	9 (28.1)	22 (25.6)	χ²=0.078	0.780
	≥50	23 (71.9)	64 (74.4)		
Glucose Concentration		2.64 (1.17,3.41)	1.96 (1.29,3.10)	Z=-0.415	0.678
Chloride Concentration		114.27 ± 8.93	116.36 ± 8.14	t=-1.207	0.230
CSF Pathogen Positivity		21 (67.7)	55 (64.7)	χ²=3.436	0.343
		1 (3.2)	0 (0.0)		
		2 (6.5)	12 (14.1)		
		7 (22.6)	18 (21.2)		
Pulmonary Pathogen Positivity		25 (78.1)	63 (72.4)	χ²=0.396	0.529
		7 (21.9)	24 (27.6)		

**Table 5:** Comparison of baseline imaging studies between the standard-dose and high-dose groups.

Indicator	Category	Standard-Dose Group (N=32)	High-Dose Group (N=87)	Statistic (χ²)	*P*
Cranial Imaging	No	18 (69.2)	71 (83.5)	2.561	0.109
	Yes	8 (30.8)	14 (16.5)		
Hydrocephalus	No	18 (69.2)	71 (83.5)	2.561	0.109
	Yes	8 (30.8)	14 (16.5)		
Abnormal Density in the Basal Cistern	No	24 (92.3)	83 (97.6)	–	0.233
	Yes	2 (7.7)	2 (2.4)		
Meningeal Enhancement	No	23 (88.5)	62 (72.9)	2.674	0.102
	Yes	3 (11.5)	23 (27.1)		
Tuberculoma	No	22 (84.6)	62 (72.9)	1.474	0.225
	Yes	4 (15.4)	23 (27.1)		
Infarction	No	19 (73.1)	64 (75.3)	0.052	0.820
	Yes	7 (26.9)	21 (24.7)		
Baseline Chest CT	Normal	1 (3.1)	1 (1.1)	2.150	0.519
	Hematogenous Disseminated Pulmonary Tuberculosis	7 (21.9)	25 (28.7)		
	Secondary Pulmonary Tuberculosis	16 (50.0)	46 (52.9)		
	Other	8 (25.0)	15 (17.2)		
Other Sites Suggestive of Tuberculosis	No	26 (81.3)	75 (86.2)	0.145	0.703
	Yes	6 (18.8)	12 (13.8)		

### 3.5 Mortality and disability outcomes between groups

Follow-up imaging results showed improvement in 26 cases (57.8%) of brain imaging and 40 cases (62.5%) of chest imaging. During the follow-up period, 40 patients in the standard-dose group and 25 patients in the high-dose group experienced disabling deaths by March, with the majority of deaths occurring at the conclusion of the intensive treatment phase. The differences in disability and mortality between the groups at the end of the 3-month intensive treatment period were statistically significant (P = 0.011). Similarly, at the 12-month follow-up, a significant difference in disability and mortality rates was observed between the two groups (P = 0.001). However, no significant differences were found in the improvement of cranial or chest imaging findings between the groups at the 3-month follow-up (P > 0.05). Detailed information is presented in **Table 6**.

**Table 6:** Comparison of imaging and prognosis at the end of the 3-month intensive phase between the standard-dose and high-dose groups.

Indicator	Category	Standard-Dose Group (N=32)	High-Dose Group (N=87)	Statistic (χ²)	*P*
Cranial Imaging Improvement	No	5 (62.5)	14 (37.8)	0.785	0.376
	Yes	3 (37.5)	23 (62.2)		
Chest Imaging Improvement	No	5 (31.2)	19 (39.6)	0.356	0.551
	Yes	11 (68.8)	29 (60.4)		
Disability or Mortality (3 Months)	No	22 (68.8)	77 (88.5)	6.530	0.011
	Yes	10 (31.3)	10 (11.5)		
Disability or Mortality (12 Months)	No	19 (59.4)	75 (86.2)	10.150	0.001
	Yes	13 (40.6)	12 (13.8)		

### 3.6 Differences in NAT2 genotype across groups with distinct INH dosage

In terms of disability and mortality rates, the standard-dose group included 32 cases, of which 13 resulted in disability or death, while the high-dose group included 87 cases, with 12 instances of disability or death. The disability and mortality rate in the high-dose group (48.0%) was significantly lower than that in the standard-dose group (52.0%), with a statistically significant difference (P = 0.001). A detailed breakdown of these findings is presented in **Table 7**. Further analysis of NAT2 genotypes revealed that within the standard-dose group, the SA genotype was associated with the lowest disability and mortality rates. In contrast, in the high-dose group, the FA genotype exhibited the lowest disability and mortality rates. However, no statistically significant differences in disability and mortality rates were observed between the FA, IA, and SA genotypes within both the standard-dose and high-dose groups. Detailed genotype-specific data can be found in **Supplementary Table 1**.

**Table 7:** Comparative analysis of different isoniazid dosages between non-disability/non-mortality and disability/mortality patients.

Indicator	Category	Standard-Dose Group (N=32)	High-Dose Group (N=87)	χ²	*P*
INH Dosage (mg)	300	19 (20.2)	13 (52.0)	10.150	0.001
	600	75 (79.8)	12 (48.0)		

### 3.7 Disability and mortality rates vary by genotype and treatment dosage

In the comparative analysis of disability and mortality rates among FA, IA, and SA genotypes, no significant differences were observed between the non-disability/non-mortality group and the disability/mortality group (P > 0.05), as detailed in **Supplementary Table 2**. When comparing disability and mortality rates between the standard-dose and high-dose groups for these genotypes, a statistically significant difference was found only in the IA type. Specifically, the disability and mortality rate in the standard-dose group (46.2%) was significantly higher than in the high-dose group (13.3%) (P = 0.019). However, no such differences were observed for the FA and SA types between the two dosage groups. Further details are provided in **Supplementary Table 3**.

## 4 Discussion

This study examines the influence of varying isoniazid (INH) dosages and NAT2 gene polymorphisms on the clinical characteristics, therapeutic approaches, and prognostic outcomes of patients with tuberculous meningitis (TBM) within the specified region. The primary objective is to elucidate the effects of INH dosage and NAT2 genetic variations on treatment efficacy, thereby providing novel insights for enhancing future therapeutic strategies and prognostic assessments.

Our findings reveal significant disparities in disability and mortality rates between patients receiving standard-dose versus high-dose INH therapy at both three-month and twelve-month intervals. Specifically, high-dose INH treatment is correlated with reduced rates of disability and mortality compared to standard dosing, with these differences achieving statistical significance (11). Notably, this research is pioneering in its exploration of the combined impact of INH dosages and NAT2 gene polymorphisms on TBM treatment outcomes within this region. The data indicate that INH dosages of 300 mg and 600 mg per day markedly influence patient prognosis, particularly regarding disability and mortality. Univariate analysis demonstrates a significant variation in disability and mortality rates among patients with the IA genotype when comparing the two dosages, whereas no such differences are observed across the broader population encompassing FA, IA, and SA genotypes. Consequently, higher INH doses are recommended for patients with the IA genotype (12).

The distribution of NAT2 gene polymorphisms among TBM patients in this region was determined to be 61.3% IA, 28.6% FA, and 10.1% SA. This distribution aligns broadly with existing literature, though regional discrepancies are evident. In Western populations, the SA genotype constitutes 40–70% of cases (6), whereas in Indian populations, the FA genotype accounts for 30–40% and the SA genotype for 60–70% (13). Domestic studies within China report IA at 40–50%, FA at 30–35%, and SA at 15–20% (14, 15). Wang Ning et al. further identified that the FA and IA genotypes predominate in the Chinese population, with the IA genotype exceeding 50%, contrasting with the genetic distributions observed in Indian and Caucasian populations but resembling those in the Japanese population, consistent with prior Chinese studies (15, 16). Our study demonstrates that high-dose INH therapy significantly diminishes disability and mortality rates in patients with the IA genotype TBM compared to standard dosing, thereby effectively reducing fatality and disability. These results corroborate previous research (16), suggesting that increased INH dosages in this genetic subgroup may enhance TBM prognosis.

Consistent with findings by Ellard et al. (17), high-dose INH therapy in this region results in lower disability and mortality rates relative to standard dosing. Ellard et al. noted that an INH dosage of 8–10 mg/kg in adults achieves cerebrospinal fluid (CSF) area under the curve (AUC) levels comparable to those in plasma observed at 5–6 mg/kg. Prior studies have indicated that the optimal INH dosing varies among populations and is intricately linked to NAT2 genotypes, which significantly influence INH plasma concentrations (18). Individuals with FA and certain IA genotypes exhibit accelerated acetylation rates under standard INH doses, resulting in reduced plasma concentrations (19–22). Conversely, those with the SA genotype metabolize INH more slowly, leading to elevated plasma drug levels (23, 24). Consequently, FA and specific IA genotype individuals necessitate higher INH dosages to attain therapeutic drug concentrations, as suboptimal levels may precipitate treatment failure or INH resistance. Conversely, excessive drug levels in SA genotype individuals may accelerate INH hydrolysis, causing amide accumulation and potential hepatotoxicity (25). The half-life of INH typically follows the sequence FA and partial IA > IA > SA, with FA and partial IA individuals achieving subtherapeutic plasma concentrations. High-dose INH therapy has been shown to improve mortality and disability rates in FA and partial IA genotype individuals, presenting a more efficacious treatment option for TBM patients (16, 26).

Ellard et al. (17) demonstrated that an INH dosage of 8–10 mg/kg in Chinese adult TBM patients achieves CSF AUC levels comparable to those in plasma at 5–6 mg/kg, underscoring the necessity of elevated INH dosages to ensure effective CSF drug concentrations. In our study, increasing the INH dosage to 600 mg/day resulted in disability and mortality rates of 13.8%, significantly lower than the 40.6% observed with the standard dose, particularly among IA genotype patients, aligning with previous studies (27). A study involving 11 Korean FA-type TBM patients suggested that a 600 mg/day INH dose may be optimal for this genotype (27). Azuma et al. (28) reported that genotype-based INH dosing reduces adverse drug reactions compared to standard dosing. In China, INH concentrations in the blood and CSF of FA-type TBM patients are approximately one-fifth to one-half of those in SA-type patients, highlighting the necessity for increased dosages in FA-type individuals. Recommended INH doses for adult TBM patients range from 600 to 1200 mg/day to achieve therapeutic plasma concentrations. Jing Wei and Cao Li (16) demonstrated that high-dose INH enhances cure rates and lowers mortality and disability rates in FA-type and partial IA-type TBM patients. Similarly, Wu et al. reported that high-dose INH significantly reduces mortality and disability in TBM patients. Chen Dili et al. found that while increasing INH doses to 600 mg/day yielded marginal improvements, further increases to 900 mg/day resulted in additional adverse effects. Zhang Yi’s research indicated that although elevating INH doses to 900 mg/day increased CSF drug concentrations, there were no significant differences in clinical improvement or CSF parameters compared to the 600 mg dose, albeit with more adverse effects. Based on this study and existing literature, a 600 mg/day INH dosage appears optimal for TBM treatment, particularly for IA-type patients, offering a balanced approach between efficacy and safety.

Furthermore, INH dosage, cranial nerve palsy, and age were identified as critical factors influencing TBM prognosis. Patients presenting with cranial nerve palsy exhibited poorer outcomes, typically characterized by restricted ocular movements, diplopia, and hearing loss. In this study, the baseline incidence of cranial nerve palsy was 17.6%, decreasing to 11% by the third month, although full recovery was not achieved in all cases. Advanced age was also associated with unfavorable outcomes, consistent with prior studies (29, 30). Additionally, persistent headaches, a common symptom in TBM, remained prevalent at the three-month mark and emerged as a significant prognostic factor (31–33). Cerebrospinal fluid analysis revealed elevated intracranial pressure and reduced glucose and chloride levels, both indicative of poor prognosis. Specifically, 75.4% of patients exhibited a CSF-to-plasma glucose ratio below 50%, and 93.2% had elevated CSF protein levels. Magnetic resonance imaging (MRI) demonstrated a high positive rate (81.85%), with frequent abnormalities including cerebral infarction (25.2%), tuberculomas (24.3%), meningeal enhancement (23.4%), and hydrocephalus (19.8%). These imaging findings are instrumental in both diagnosis and therapeutic evaluation.

## 5 Conclusion

In conclusion, this study underscores the significant role of INH dosage and NAT2 gene polymorphisms in determining the clinical outcomes of TBM patients. High-dose INH therapy, particularly in individuals with the IA genotype, offers a promising approach to improving prognosis by reducing disability and mortality rates. Additionally, factors such as cranial nerve palsy, age, persistent headaches, and specific CSF and MRI findings are pivotal in predicting TBM outcomes. These insights collectively inform more personalized and effective treatment strategies for TBM.

## Data Availability

The original contributions presented in the study are included in the article/**Supplementary Material**. Further inquiries can be directed to the corresponding authors.
