# Supplementary material for: The role of isoniazid dosage and NAT2 gene polymorphism in the treatment of tuberculous meningitis
Source: Front Immunol. 2025 Jan 20;15:1535447. doi: 10.3389/fimmu.2024.1535447 (PMC11788379; doi:10.3389/fimmu.2024.1535447)
Supplement: Supplementary file 1 [file Table1.docx]

**Supplementary Materials**

**Supplementary Table 1** Comparative Analysis of Disability and Mortality Rates Among FA, IA, and SA Types Under Different INH Dosages

| **INH Dosage** | **NAT2 Genotype** | **Non-Disability/Non-Mortality Patients (%)** | **Disability/Mortality Patients (%)** | ***P*** |
| --- | --- | --- | --- | --- |
| 300mg | FA | 10(62.5) | 6(37.5) | 0.873 ***^c^*** |
|  | IA | 7(53.8) | 6(46.2) |  |
|  | SA | 2(66.7) | 1(33.3) |  |
| 600mg | FA | 16(88.9) | 2(11.1) | 0.779 ***^c^*** |
|  | IA | 52(86.7) | 8(13.3) |  |
|  | SA | 7(77.8) | 2(22.2) |  |

Notes: c indicates Fisher's exact test was used for statistical analysis.

**Supplementary Table 2.** Comparative Analysis of Disability and Mortality Rates Among FA, IA, and SA Types Between Two Groups

| **NAT2 Genotype** | **Non-Disability/Non-Mortality Patients (%)** | **Disability/Mortality Patients (%)** | **χ²** | ***P*** |
| --- | --- | --- | --- | --- |
| FA | 26(76.5) | 8(23.5) | 0.393 | 0.822 |
| IA | 59(80.8) | 14(19.2) |  |  |
| SA | 9(75.0) | 3(25.0) |  |  |

**Supplementary Table 3.** Comparative Analysis of Disability and Mortality Rates Among FA, IA, and SA Types Between the Standard-Dose and High-Dose Groups

| **NAT2 Genotype** | **INH Dosage** | **Non-Disability/Non-Mortality Patients (%)** | **Disability/Mortality Patients (%)** | ***P*** |
| --- | --- | --- | --- | --- |
| FA | 300 | 10(62.5) | 6(37.5) | 0.110 ***^c^*** |
|  | 600 | 16(88.9) | 2(11.1) |  |
| IA | 300 | 7(53.8) | 6(46.2) | 0.019 |
|  | 600 | 52(86.7) | 8(13.3) |  |
| SA | 300 | 2(66.7) | 1(33.3) | >0.999***^c^*** |
|  | 600 | 7(77.8) | 2(22.2) |  |
